# Urine proteomics of primary membranous nephropathy using nanoscale liquid chromatography tandem mass spectrometry analysis

**DOI:** 10.1186/s12014-018-9183-3

**Published:** 2018-02-07

**Authors:** Lu Pang, Qianqian Li, Yan Li, Yi Liu, Nan Duan, Haixia Li

**Affiliations:** 1 Department of Clinical Laboratory, Peking University First Hospital, Beijing, China; 2 Laboratory of Interdisciplinary Research, Institute of Biophysics, Chinese Academy of Sciences, Beijing, China

**Keywords:** Primary membranous nephropathy, Urine, Proteomics, Alpha-1-antitrypsin, Afamin

## Abstract

**Background:**

Primary membranous nephropathy (PMN) is an important cause of nephrotic syndrome in adults. Urine proteome may provide important clues of pathophysiological mechanisms in PMN. In the current study, we analyzed and compared the proteome of urine from patients with PMN and normal controls.

**Methods:**

We performed two technical replicates (TMT1 and TMT2) to analyze and compare the urine proteome from patients with PMN and normal controls by tandem mass tag (TMT) technology coupled with nanoscale liquid chromatography tandem mass spectrometry analysis (LC–MS/MS). Gene ontology (GO) enrichment analysis was performed to analyse general characterization of the proteins. The proteins were also matched against the database of Kyoto Encyclopedia of Genes and Genomes (KEGG). For validation, Western blot was used to analyze the selected proteins.

**Results:**

A total of 509 proteins and 411 proteins were identified in TMT1 and TMT2, respectively. 249 proteins were both identified in two technical replicates. GO analysis and KEGG analysis revealed immunization and coagulation were predominantly involved. Among the differential protein, the overexcretion of alpha-1-antitrypsin (A1AT) and afamin (AFM) were validated by Western blot analysis.

**Conclusions:**

Our data showed the important role of immunologic mechanism in the development of PMN, and the value of urinary A1AT and AFM in biomarker discovery of patients with PMN. The discovery of the overexcretion of A1AT and AFM in the urine can help to further elucidate pathogenetic mechanisms involved in PMN.

**Electronic supplementary material:**

The online version of this article (10.1186/s12014-018-9183-3) contains supplementary material, which is available to authorized users.

## Background

Primary membranous nephropathy (PMN) is the most common cause of nephrotic syndrome in the adult population [1, 2] and it is one of the most common diseases affecting the glomerulus [2]. The kidney filtration barrier consists of capillary endothelial cells, glomerular basement membrane and highly specialized epithelial cell, the podocytes [3, 4]. In patients with PMN, this filtration barrier is injured, leading to massive loss of proteins in urine (proteinuria) [5]. Histopathologically, this disorder is characterized by discovery of immune complexes deposits in a specific part of glomerulus between the glomerular basement membrane and podocyte (called subepithelium), which contain podocyte antigens or planted antigens and circulating antibodies specific to those antigens, resulting in complement activation [2]. This discovery corroborates the hypothesis that the most likely pathogenesis of PMN is autoimmune.

Tremendous insights into the pathophysiology of PMN have been made recently, with studies that have identified M-type phospholipase A2 receptor (PLA2R) [6] and thrombospondin type-1 domain-containing 7A (THSD7A) [7] as two major autoantigens in PMN. These finding supported the theory that podocytes act as sources of antigens for the formation of subepithelial immune complexes deposits [8]. Serum anti-PLA2R antibody identifies approximately 60–80% of cases of PMN [9–11]. Despite the undeniable potential of anti-PLA2R antibody as a biomarker of disease in patients with PMN, this antibody does not explain the etiology of the disease in a substantial proportion of cases [12].

Despite progress in the understanding of pathogenesis, the diagnosis of PMN is definitively dependent on only renal biopsy [13]. The exact pathogenesis of PMN remains unknown, but podocytes and podocytes-related proteins appear to have a pivotal role in the development of PMN. The pathogenicity of anti-PLA2R has not yet been confirmed [14]. However, human anti-THSD7A has recently been shown to induce PMN with proteinuria in mice [15]. The morphology of healthy podocyte foot processes is necessary for maintaining the characteristics of the kidney filtration barrier [16]. Interaction of circulating autoantibodies with antigens at the podocyte cell membrane-basement membrane interface generally is regarded as the fundamental pathological mechanism [17]. Since the visceral epithelial cell of Bowman’s capsule is podocytes, the content of podocytes will be released into urine when podocytes were destroyed by membrane attack complex. Therefore, urine can be considered as a potential source to provide important clues of pathophysiological mechanisms of PMN.

Nanoscale liquid chromatography tandem mass spectrometry analysis (LC–MS/MS) may be considered a novel method for identifying candidate biomarkers for patients suffering from PMN [18]. Tandem mass tag (TMT) is a label-based quantification technology that enables accurate and simultaneous comparison of multiple samples for protein and peptide quantification [19]. TMT coupled with LC–MS/MS has the ability to analyze hundreds of proteins with the high-resolution, mass accuracy, and sensitivity [20]. LC–MS/MS has been applied in the proteomic analysis of various kidney diseases, such as acute kidney injury [21], lupus nephritis [22], diabetic nephropathy [23] and IgA nephropathy [24]. However, proteomic analysis of PMN has been rarely studied, especially in urine.

Our aim was to test urine proteomics as a non-invasive method for identification of new protein biomarkers of PMN in urine, and link them to pathogenesis of the disease through known signaling and metabolic pathways. In this study, we performed proteomic analysis using TMT technology coupled with LC–MS/MS, comparing PMN urine samples with normal control groups. The proteomic results were validated with Western blot, providing the preliminary evidence of protein biomarkers in urine of PMN patients.

## Methods

### Sample collection

Midstream morning urine samples were collected from patients of biopsy proven PMN with positive anti-PLA2R antibody (Group A, n = 32) and negative anti-PLA2R antibody (Group B, n = 31). Patient characteristics are shown in Additional file 1: Table S1 and Additional file 2: Table S2. Cases of secondary MN were excluded from the present study, in particular patients with systemic autoimmunity diseases, viral hepatitis B and C and HIV infection, neoplastic conditions and exposure to toxic agents. All samples were collected at the time of biopsy. Urine samples from healthy volunteers (Group C, n = 32) were also collected (Additional file 3: Table S3). Samples were kept for less than 4 h at room temperature followed by low speed centrifugation at 2000×*g*, 10 min at room temperature to remove cellular debris and then stored at − 80 °C until use.

At first, we performed the first TMT experiment (called TMT1 in this study). To increase accuracy repeatability, we performed another TMT experiment (called TMT2 in this study) by increasing the sample size and replicates to validate TMT1 (Table 1). Samples from group A, group B and group C (9 patients in each group) were tested by Western blot (Additional file 1: Table S1, Additional file 2: Table S2, Additional file 3: Table S3).

**Table 1:** Flow chart of experimental design with three biological replicates and two technical replicates

Replicates	Groups	Number of samples	TMT tags
TMT1	Group A	5	126
	Group B	4	130
	Group C	5	131
TMT2			
TMT2a	Group A	9	126
	Group B	9	127C
	Group C	9	127 N
TMT2b	Group A	9	128 N
	Group B	9	129C
	Group C	9	130C

This study was approved by the ethics committee of Peking University First Hospital and informed consents were obtained from all participants.

### Protein precipitation

The 10 mL sample aliquots from each participant were thawed, and 2.5 mL of trichloroacetic acid (TCA) precipitation solution (30% v/v) were added to the sample aliquots to a final TCA concentration of 6% v/v. Strong vortexing for 1 min and overnight incubation at − 20 °C were performed. The mixtures were centrifuged at 14,000×*g* for 15 min at 4 °C and the supernatants were discarded. In order to eliminate traces of acid that can negatively affect the digestion efficiency, the pellets were resuspended in 2.5 mL of chilled acetone (− 20 °C) and clarified by centrifugation at 14,000×*g* at 25 °C for 10 min. The wash step was repeated once more. The supernatants were discarded and the pellets were dried naturally. The dry pellets were resuspended in 300 μL solubilization buffer (8 mol/L urea and 0.1 mol/L ammonium bicarbonate), vortexed strongly for 1 min and incubated for 15 min at 37 °C. Protein concentration was determined using the bicinchoninic acid protein assay.

### Protein preparation

We pooled the samples from each participant on equal quantity to form three pooled samples in TMT1 and six pooled samples in TMT2 (Table 1). 200 μL reducing solution [10 mg/mL dithiothreitol (DTT)] was added and incubate for 1 h at 37 °C. Then, 200 μL alkylation solution [12 mg/mL iodoacetamide (IAA)] was added to block reduction of cysteine residues and incubated for 1 h in the dark. The DTT, IAA and other low-molecular-weight components were removed using trypsin buffer (1 mmol/L CaCl_2_ and 100 mmol/L Tris–HCl, pH 8.0) by repeated ultrafiltration (3 kD Microcon; Millipore Corp., Billerica, MA, USA). The samples were then digested with trypsin with the ratio of protein:trypsin = 100:1 at 37 °C overnight.

### TMT labeling

Protein peptides (50 μg) from each group were processed strictly according to the manufacturer’s protocol for TMT Mass Tag Labeling Kits and Reagents (Thermo Fisher Scientific, Waltham, MA, USA). The protein peptides were labeled randomly with 126, 130, 131 TMTsixplex tags for TMT1 and 126, 127C, 127N, 128N, 129C, 130C TMT10plex for TMT2 (Table 1). The TMT Label Reagents were equilibrated to room temperature and reconstituted with 41 μL of anhydrous acetonitrile. The reagents were dissolved for 5 min with occasional vortexing and then centrifuged to gather the solution. The TMT Label Reagents were added to the corresponding peptide samples and incubated the reaction for 1 h at room temperature. 8 μL of 5% hydroxylamine were added to the sample and incubate for 15 min to quench the reaction. Samples from three groups of TMT1 and six groups of TMT2 were mixed equally and lyophilized, respectively. 1 μL aliquot of sample was removed from each group to test labeling and extraction efficiency, and the sample was subjected to a matrix assisted laser desorption ionization procedure after Ziptip desalting.

### Strong cationic exchange chromatography

The mixed peptides were dissolved in buffer A (2% acetonitrile (ACN) and 20 mmol/L ammonium formate, pH 10.0). Then, the samples were loaded onto a reverse-phase column (Luna C18, 4.6 × 150 mm; Phenomenex, Torrance, CA, USA) and eluted using a step linear elution program: 0–10% buffer B (500 mmol/L KCl, 10 mmol/L KH_2_PO_4_ in 25% ACN, pH 2.7) for 10 min, 10–20% buffer B for 25 min, 20–45% buffer B for 5 min and 50–100% buffer B for 5 min at a flow rate of 0.7 mL/min. The samples were collected each min and centrifuged for 5–45 min. The fractions (about 40) collected were finally combined into 10 pools and desalted on C18 Cartridges (Empore™ standard density SPE C18 Cartridges, bed I.D. 7 mm, 3 mL volume; Sigma, St. Louis, MO, USA).

### LC–MS/MS analysis

The reconstituted peptides were analyzed with the Q-Exactive mass spectrometer (Thermo Fisher Scientific, Waltham, MA, USA) coupled with a nano high-performance liquid chromatography (UltiMate 3000 LC Dionex; Thermo Fisher Scientific, Waltham, MA, USA) system. The peptides were loaded onto a C18 reversed phase column (3 μm C18 resin, 75 μm × 15 cm) and separated on an analytical column (5 μm C18 resin, 150 μm × 2 cm; Dr. Maisch GmbH, Ammerbuch, Germany) using mobile phase A: 0.5% formic acid (FA)/H_2_O and B: 0.5% FA/ACN at a flow rate of 300 nL/min, using a 150 min gradient. Spectra were acquired in data-dependent mode. The 10 most intense ions were selected for MS scanning (300–1800 m/z, 60,000 resolution at 400 m/z, accumulation of 1 × 10^6^ ions for a maximum of 500 ms, 1 microscan). The isolation window was 1.3 m/z and the MS/MS spectra were accumulated for 150 ms using an Orbitrap. MS/MS spectra were measured at resolution of 15,000 at 400 m/z. Dynamic precursor exclusion was allowed for 2 min after each MS/MS spectrum measurement and was set to 17,500 at 200 m/z. Normalized collision energy was 30 eV and the underfill ratio, which specifies the minimum percentage of the target value likely to be reached at the maximum fill time, was defined as 0.1%. The instrument was run with peptide recognition mode enabled. All of the MS proteomics data have been deposited to the integrated proteome resources [25] with the accession number IPX0001113000.

### Sequence database search and data analysis

The raw mass data are processed for the peptide data analysis using Proteome Discoverer (v 1.4.0.288; Thermo Fisher Scientific, Waltham, MA, USA) with a false discovery rate (FDR) < 1% and expected cutoff or ion score < 0.05 (with 95% confidence) for searching the UniProt Human Complete Proteome database: digestion with trypsin with no more than two missed cleavages, TMT modification to lysine side chains and N-termini, and variable oxidation of methionine residues. A mass tolerance of 10 ppm for intact peptide masses and 0.6 Da for fragmented ions were permitted. All peptide spectra scoring higher against a reversed human sequence were eliminated from further consideration. Protein probabilities were assigned using the Protein Prophet algorithm and proteins with at least one unique peptide were identified. The upregulated or downregulated proteins in three replicates with relative quantification 1.5 fold-changes were selected as being differentially expressed in the data.

### Bioinformatics analyses

We performed gene ontology (GO) bioinformatics analysis on the differentially expressed proteins with a 1.5-fold change to catalog the molecular functions, cellular components and biological processes [26]. The enrichment analysis of the proteins was conducted with all proteins GO biological processes database information from our study using the following formula [27]:

$$ P = 1\sum\limits_{i = 0}^{m - 1} {\frac{{\left( {\begin{array}{*{20}c} M \\ i \\ \end{array} } \right)\left( {\begin{array}{*{20}c} {N - M} \\ {n - i} \\ \end{array} } \right)}}{{\left( {\begin{array}{*{20}c} N \\ n \\ \end{array} } \right)}}} $$

where N is the number of all proteins within the GO annotation information, n is the number of differentially regulated proteins within GO annotation information, M is the number of proteins for a given GO annotation, and m is the number of differentially regulated proteins following a given GO annotation. Significant GO enrichment was defined as *P* ≤ 0.05.

The interactions among these proteins regarding the biological pathways were determined using Kyoto Encyclopedia of Genes and Genomes (KEGG) database to better understand these differentially proteins in relation to the published literature. The Pathway Maps tool was used to enrich the pathways with all proteins KEGG database information from our study and *P* values were calculated based on a hypergeometric distribution. Significant pathway enrichment was defined as *P* ≤ 0.05.

### Western blot analysis

The increases of alpha-1-antitrypsin (A1AT) and afamin (AFM) were validated by Western blot. For internal validation, due to the inadequate protein, we only validated with 13 samples of Group A, 13 samples of Group B and 10 samples of Group C. For external validation, nine samples for each group were validated. The datail characteristics of the samples are shown in Additional file 1: Table S1, Additional file 2: Table S2 and Additional file 3: Table S3. The protein samples were resolved by sodium dodecyl sulfate polyacrylamide gel electrophoresis, transferred onto polyvinylidene fluoride membranes and blocked with 5% nonfat milk at room temperature for 1 h. Mouse monoclonal antibody against A1AT (1:1000, ab9399; Abcam, Cambridge, MA, USA) and mouse monoclonal antibody against AFM (1:500, sc-373849; Santa Cruz Biotechnology, Santa Cruz, CA, USA) were used as primary antibody. Goat anti-mouse horse radish peroxidase (HRP)-conjugated IgG (1:5000, sc-2005; Santa Cruz Biotechnology, Santa Cruz, CA, USA) was used as secondary antibody. Signals were developed using Immobilon Western HRP substrate (WBKLS0100; Millipore, Billerica, MA, USA).

### Statistical analysis

Statistical analyses were performed using the SPSS software version 19.0 for Windows (IBM, Chicago, IL, USA). Graphs were prepared using GraphPad Prism version 6.0 (GraphPad Software, San Diego, CA, USA). Values are represented as means ± standard deviations. Student *t* test was used to compare differences between continuous data. A two-tailed *P* value < 0.05 was considered to be statistically significant.

## Results

GO analysis and KEGG analysis showed immunization and coagulation were predominantly involved.

A total of 509 proteins and 411 proteins were identified in TMT1 and TMT2, respectively (Additional file 4: Table S4, Additional file 5: Table S5). 249 proteins were identified in both TMT1 and TMT2.

**Fig. 1:**
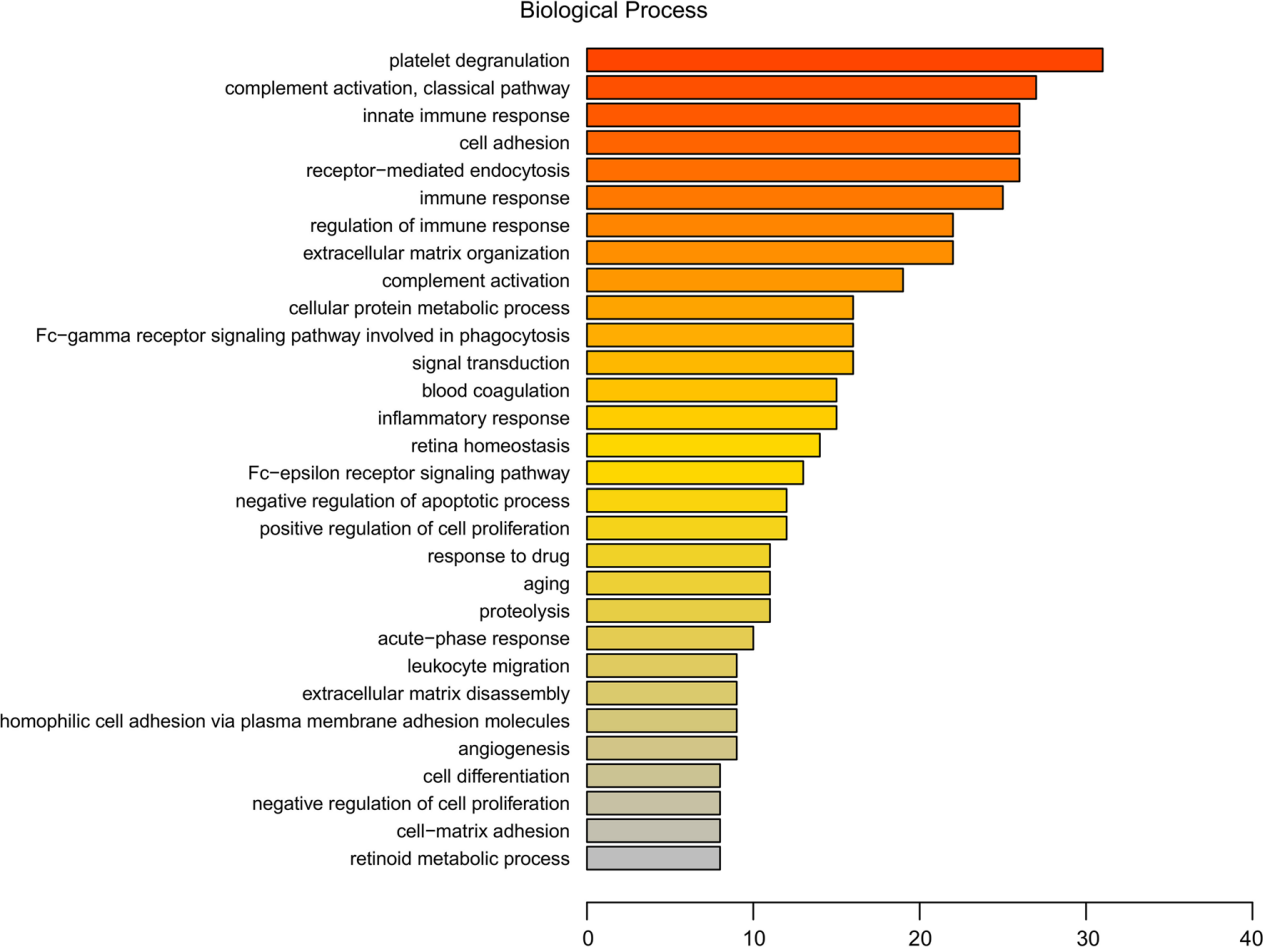
Gene Ontology (GO) annotation analysis of biological process of 249 identified proteins. Abscissa representatives the number of proteins

**Fig. 2:**
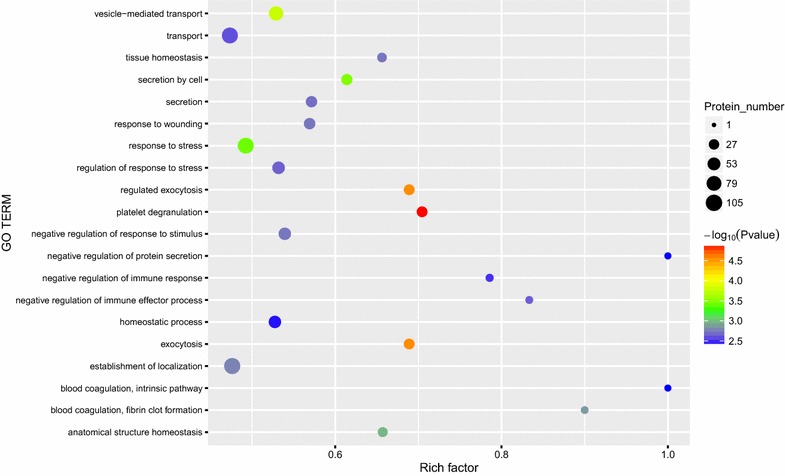
Gene Ontology (GO) enrichment analysis of 249 identified proteins in biological processes. Bubble diagram of the over-represented GO terms

We performed GO analysis to enrich and cluster the 249 differential proteins. It was revealed that most of the proteins were involved in the platelet degranulation (n = 31), complement activation by classical pathway (n = 27), innate immune response (n = 26), cell adhesion (n = 26), receptor-mediated endocytosis (n = 26) and immune response (n = 25) (Fig. 1). GO enrichment analysis of biological processes showed that platelet degranulation (*P* < 0.05) is the main GO term associated with these proteins (Fig. 2). The detail information of GO enrichment of molecular functions, cellular components and biological processes was shown in Additional file 6: Table S6.

**Fig. 3:**
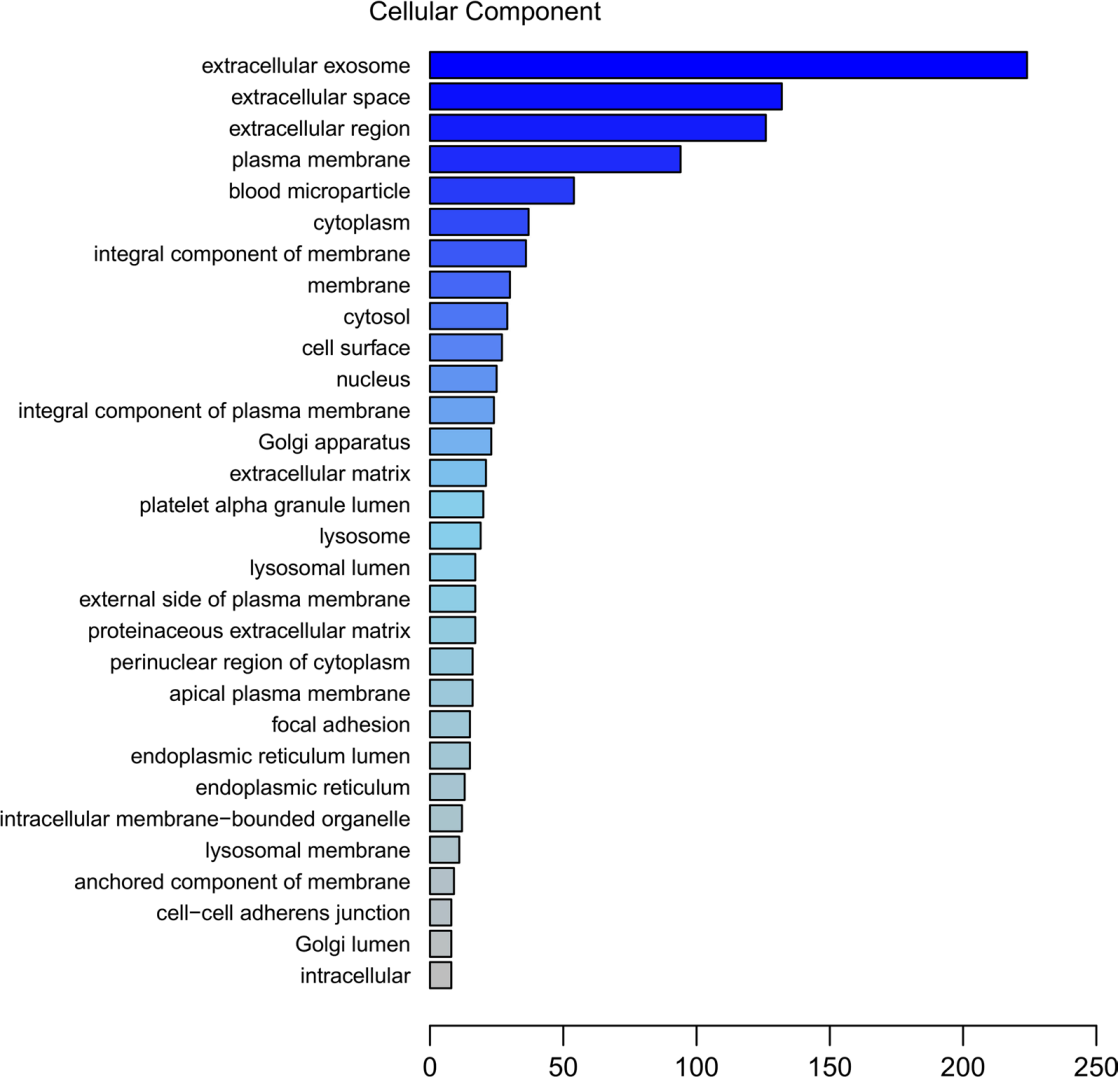
Gene Ontology (GO) annotation analysis of cellular component of 249 identified proteins. Abscissa representatives the number of proteins

**Fig. 4:**
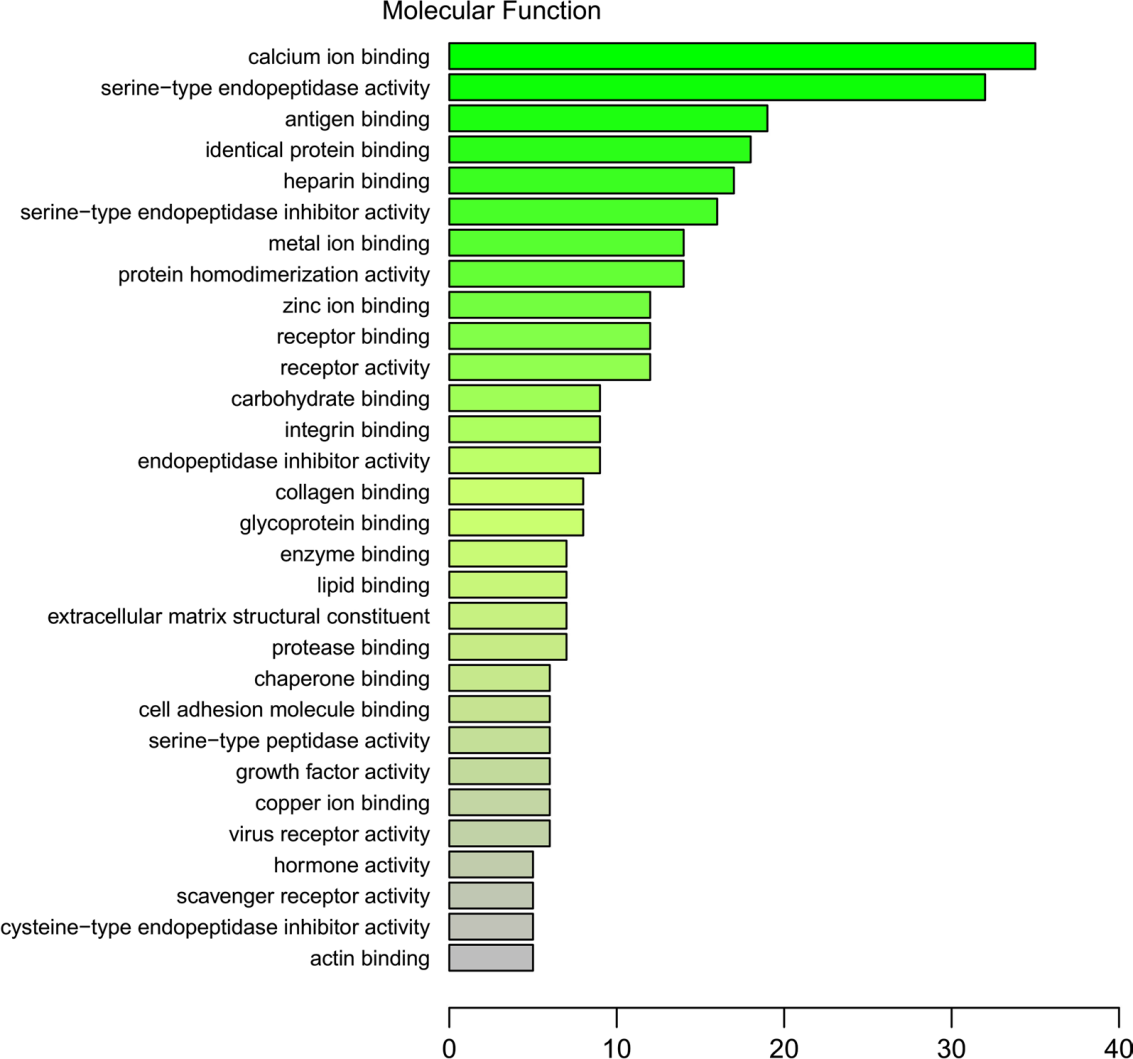
Gene Ontology (GO) annotation analysis of molecular function of 249 identified proteins. Abscissa representatives the number of proteins

In addition, GO annotation analysis revealed that the subcellular proteins were distributed in the extracellular exosome (n = 224), extracellular space (n = 132), extracellular region (n = 126) and plasma membrane (n = 94) (Fig. 3) and associated with calcium ion binding (n = 35), serine-type endopeptidase activity (n = 32), antigen binding (n = 19) and identical protein binding (n = 18) (Fig. 4).

**Fig. 5:**
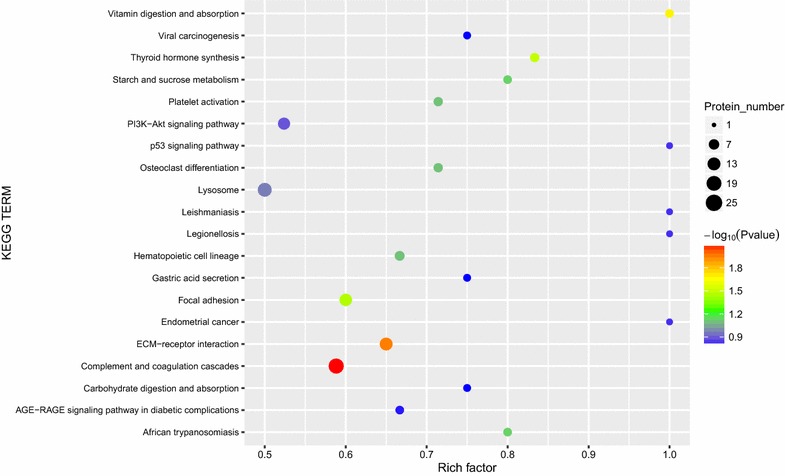
Kyoto Encyclopedia of Genes and Genomes (KEGG) enrichment analysis of 249 identified proteins. Bubble diagram of the over-represented KEGG pathways

**Fig. 6:**
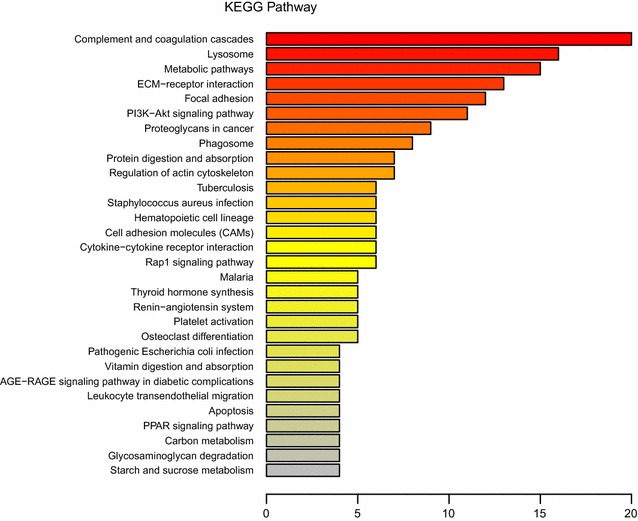
Kyoto Encyclopedia of Genes and Genomes (KEGG) annotation analysis of 249 identified proteins. Abscissa representatives the numbers of proteins

**Fig. 7:**
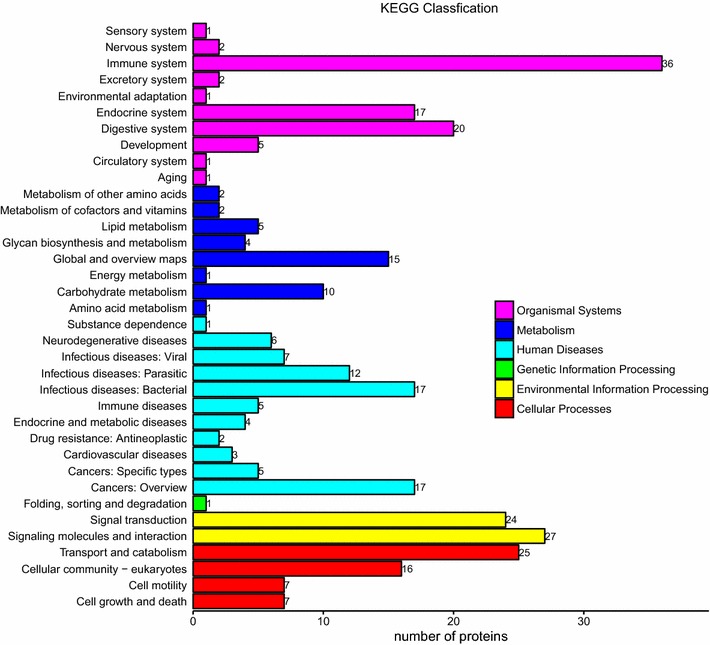
Kyoto Encyclopedia of Genes and Genomes (KEGG) function classification analysis of 249 identified proteins

The KEGG pathway annotation analysis indicated that complement and coagulation cascades (n = 20, *P* < 0.05) is the main KEGG pathway associated with these proteins (Figs. 5 and 6). KEGG function classification analysis revealed that immune system (n = 36) and signaling molecules and interaction (n = 27) were predominantly involved (Fig. 7). The detail information of KEGG pathway enrichment was shown in Additional file 7: Table S7.

### AFM and A1AT were increased in urine from patients with PMN

**Fig. 8:**
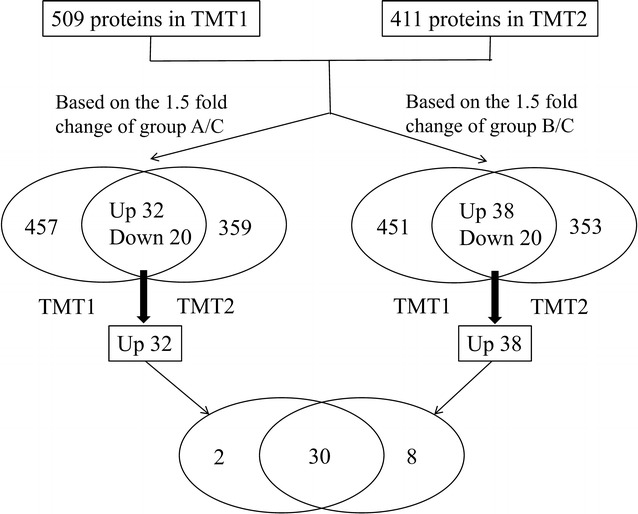
Flow chart of determination target protein alpha-1-antitrypsin (A1AT) and afamin (AFM) in TMT1 and TMT2

Intersecting the set of differential proteins in the TMT1 and TMT2, 32 proteins and 38 proteins were found to be upregulated based on the 1.5 fold-changes of group A/C and group B/C respectively. The upregulated proteins were highly consistent in both groups, resulting in 30 proteins identified by both group A/C and group B/C (Fig. 8). The top 10 proteins of mean fold change were listed in Table 2. The maximum differentially abundant protein enriched in both groups was alpha-1-antitrypsin (A1AT), a serine proteinase inhibitor. In addition, we selected afamin from the top 10 proteins for further analysis.

**Table 2:** Top 10 of 30 proteins determined by both group A/C and group B/C with the high scores of mean fold change when intersecting the set of differential proteins in the TMT1 and TMT2

No.	Protein ID	Protein name	Gene name	Mean fold change
1	P01009	Alpha-1-antitrypsin	SERPINA1	14.508
2	P02787	Serotransferrin	TF	14.082
3	P00450	Ceruloplasmin	CP	12.532
4	P02768	Serum albumin	ALB	8.734
5	P00915	Carbonic anhydrase 1	CA1	8.606
6	P02766	Transthyretin	TTR	8.541
7	P00738	Haptoglobin	HP	8.415
8	P02750	Leucine-rich alpha-2-glycoprotein	LRG1	7.424
9	P04217	Alpha-1B-glycoprotein	A1BG	7.065
10	P43652	Afamin	AFM	6.954

### The increase of A1AT and AFM were validated by Western blot

**Fig. 9:**
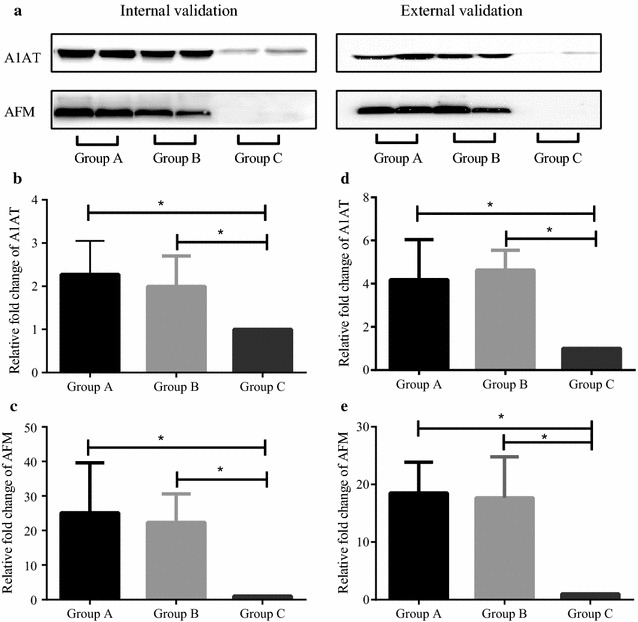
Overexcretion of alpha-1-antitrypsin (A1AT) and afamin (AFM) in urine from patients with PMN. **a** Representative images of Western blot. Bar chats of relative fold-change of A1AT (**b**) and AFM (**c**) of internal validation, and A1AT (**d**) and AFM (**e**) of external validation. Asterisk denotes *P* < 0.05 using student *t* test. The bars represent the means ± standard deviations

The findings of the proteomic analysis were validated by Western blot. As shown in Fig. 9, A1AT and AFM were significantly increased in PMN urine compared to control samples.

## Discussion

Urine proteome may provide important clues of pathophysiological mechanisms in PMN because urine samples are kidney-derived and easily acquirable. The expression of lysosome membrane protein-2 in urinary microvesicles has been suggested to be a biomarker for PMN [28], but this study only focused on urinary microvesicles. When podocytes, one of the layers of filtration barrier, were destroyed through the way of complement mediated cytolysis, the cell lysate will be released into urine. In the present study, we demonstrate the value of urine in biomarker discovery in PMN. Comparison of the urinary proteome of patients with PMN to normal controls enabled identification of new proteins possibly involved in the pathogenesis of PMN. This pilot study confirmed that the application of our approach is successful in identifying proteins upregulated in PMN and found that A1AT and AFM were significantly increased in PMN urine compared to control samples.

By GO function analysis and KEGG pathway analysis, our study demonstrated immunization was predominantly involved, including complement activation and immune response found in biological process analysis and complement cascades and immune system found in pathway analysis. It has been clarified PMN is driven by an underlying immune response [2]. The immune complexes cause podocyte injury characterized by actin–cytoskeleton disorganization and podocyte foot-process effacement, leading to increased permeability of the glomerular filtration barrier and eventually to massive proteinuria and nephrotic syndrome [29]. In addition, our study discovered that coagulation was also involved, including platelet degranulation found in biological process analysis and coagulation cascades found in pathway analysis. PMN is the pathological type of nephrotic syndrome associated with the highest incidence of thromboembolic events [30]. The underlying mechanisms of PMN-related hypercoagulability are not fully understood [31] and greater efforts should be made to clarify whether platelet degranulation was involved in PMN-related thromboembolic events.

A1AT, also named α1-Pi (α1 proteinase inhibitor) and SERPINA1 (serine protease inhibitor, group A, member 1), is the most abundant serum serine protease inhibitor [32]. Its main physiological role is to inhibit the activity of different endogenous serine proteases, such as elastase, myeloperoxidase and proteinase-3 [32, 33]. Due to its anti-protease activity, A1AT can exert tissue-protective effects. A1AT levels are upregulated in the renal tissue of adenine-induced chronic renal failure model [34] and in vivo administration of clinical grade A1AT improves renal function, decreases acute tubular necrosis and ameliorates acute kidney injury following experimental kidney ischemia reperfusion damage [35]. The overexcretion of A1AT have been described in the urine of IgA nephropathy, membranoproliferative glomerulonephritis, minimal change disease, focal segmental glomerulosclerosis and membranous nephropathy [36, 37]. Upregulation of A1AT could lead to inhibition of neutrophil elastase, which can contribute to accumulation of mesangial matrix, maintaining the elasticity of blood vessels and glomerular integrity [34]. A1AT is located in the cytoplasm of podocytes within sclerotic glomeruli [13] and its relationship with podocyte dysfunction and podocyte stress need to be further investigated.

AFM is a glycoprotein that is present in biological fluids such as plasma, urine, cerebrospinal, ovarian follicular and seminal fluids [38]. It is the fourth member of the human albumin gene family, which includes albumin, α-fetoprotein and vitamin D-binding protein [39]. Comparative proteomics have identified AFM as a potential urine biomarker for focal segmental glomerulosclerosis [40], paediatric idiopathic nephrotic syndrome [41] and IgA nephropathy [42]. Further studies in larger patient population are needed to investigate the role of AFM in kidney diseases.

This study has several limitations. To increase accuracy and reduce variability, we performed TMT2 by increasing the sample size and replicates to validate TMT1, resulting in the mismatch of of biological samples added into the pool. We did not assess other clinically pathological pattern of glomerular diseases, thus we did not clearly confirm the effect of differential diagnosis of A1AT and AFM. In addition, we only validated the candidates proteins in a small sized sample set by Western blot, clinical usefulness need to be verified with large sample size.

## Conclusions

In conclusion, we succeeded in performing urine proteomics of PMN using TMT technology coupled with LC–MS/MS. According to the GO and KEGG analyses, it was demonstrated that these proteins are involved in multiple biological processes and pathways, but mainly in immune response and coagulation cascades. Interestingly, A1AT and AFM exhibited significantly changes and verified by Western blot. Further research is needed to explore the role of these protein as the candidate biomarkers of PMN and functions in pathogenesis.

## Additional files

**Additional file 1: Table S1.** Clinical characteristics of patients in group A.

**Additional file 2: Table S2.** Clinical characteristics of patients in group B.

**Additional file 3: Table S3.** Clinical characteristics of healthy volunteers in group C.

**Additional file 4: Table S4.** Raw data of the information of 509 proteins in TMT1.

**Additional file 5: Table S5.** Raw data of the information of 411 proteins in TMT2.

**Additional file 6: Table S6.** The detail information of gene ontology (GO) enrichment analysis.

**Additional file 7: Table S7.** The detail information of Kyoto Encyclopedia of Genes and Genomes (KEGG) pathway enrichment analysis.

## Abbreviations

PMN: primary membranous nephropathy
GO: gene ontology
KEGG: Kyoto Encyclopedia of Genes and Genomes
A1AT: alpha-1-antitrypsin
AFM: afamin
PLA2R: M-type phospholipase A2 receptor
THSD7A: thrombospondin type-1 domain-containing 7A
TMT: tandem mass tag
LC–MS/MS: liquid chromatography coupled with tandem mass spectrometry
TCA: trichloroacetic acid
DTT: dithiothreitol
IAA: iodoacetamide
ACN: acetonitrile
FA: formic acid
HRP: horse radish peroxidase
SH2: src homology 2
SH3: src homology 3
α1-Pi: α1 proteinase inhibitor
SERPINA1: serine protease inhibitor, group A, member 1
