# Supplementary material for: Urine proteomics of primary membranous nephropathy using nanoscale liquid chromatography tandem mass spectrometry analysis
Source: Clin Proteomics. 2018 Feb 7;15:5. doi: 10.1186/s12014-018-9183-3 (PMC5801694; doi:10.1186/s12014-018-9183-3)
Supplement: Supplementary file 1 — Additional file 1: Table S1. Clinical characteristics of patients in group A. [file 12014_2018_9183_MOESM1_ESM.docx]

**Table S1. Clinical characteristics of patients in group A.**

|  | Gender | Age (years) | Proteinuria (g/24h) | Serum creatinine (μmol/L) | eGFR (ml/min/1.73m^2^) | Urea (mmol/L) | Serum anti-PLA2R antibody (RU/mL) | For validation |
| --- | --- | --- | --- | --- | --- | --- | --- | --- |
| *TMT1* | | | | | | | |  |
|  | M | 64 | 6.03 | 97.7 | 70.168 | 5.88 | 277.00 | No |
|  | M | 47 | 9.28 | 70.1 | 108.276 | 7.56 | 55.00 | No |
|  | M | 52 | 1.47 | 59.4 | 110.342 | 5.75 | 129.00 | No |
|  | F | 75 | 5.26 | 70.7 | 72.376 | 6.74 | 101.00 | No |
|  | M | 28 | 3.85 | 86.0 | 105.422 | 3.40 | 36.00 | No |
| *TMT2a* | | | | | | | |  |
|  | F | 50 | 0.83 | 42.4 | 114.773 | 3.62 | 30.00 | No |
|  | M | 44 | 7.50 | 78.9 | 103.866 | 8.90 | 21.00 | Yes |
|  | M | 53 | 3.03 | 64.5 | 105.922 | 5.90 | 56.00 | Yes |
|  | M | 73 | 4.11 | 69.5 | 89.257 | 4.10 | 24.00 | Yes |
|  | M | 56 | 20.03 | 81.3 | 92.687 | 9.60 | 459.00 | No |
|  | M | 31 | 4.80 | 83.0 | 107.751 | 4.37 | 88.00 | Yes |
|  | M | 24 | 7.29 | 79.1 | 119.409 | 3.13 | 378.00 | Yes |
|  | M | 39 | 14.33 | 63.3 | 117.774 | 4.70 | 41.00 | Yes |
|  | M | 65 | 2.03 | 68.6 | 94.924 | 7.50 | 57.00 | Yes |
| *TMT2b* | | | | | | | |  |
|  | M | 56 | 3.13 | 57.6 | 108.650 | 4.13 | 44.00 | Yes |
|  | M | 70 | 2.40 | 75.6 | 88.060 | 5.40 | 57.00 | Yes |
|  | M | 34 | 3.79 | 72.9 | 115.106 | 5.57 | 82.00 | Yes |
|  | M | 62 | 2.81 | 84.3 | 85.052 | 5.90 | 59.00 | No |
|  | F | 46 | 1.24 | 57.3 | 106.909 | 4.21 | 63.00 | No |
|  | F | 50 | 4.07 | 33.6 | 123.902 | 5.72 | 93.00 | Yes |
|  | M | 68 | 2.39 | 102.8 | 64.152 | 7.00 | 60.00 | No |
|  | M | 25 | 11.05 | 88.1 | 104.572 | 4.80 | 196.00 | Yes |
|  | F | 39 | 3.88 | 61.0 | 110.009 | 4.50 | 491.00 | Yes |
| *Western blot* | | | | | | | |  |
|  | M | 48 | 2.92 | 129.6 | 55.793 | 5.35 | 46.00 | Yes |
|  | F | 48 | 3.36 | 81.0 | 74.225 | 4.80 | >1500.00 | Yes |
|  | M | 67 | 3.66 | 95.4 | 70.711 | 6.60 | 78.00 | Yes |
|  | F | 64 | 4.14 | 64.6 | 87.202 | 6.24 | 80.00 | Yes |
|  | M | 16 | 13.11 | 85.3 | 79.464 | 6.00 | 40.00 | Yes |
|  | F | 58 | 4.10 | 53.8 | 100.326 | 5.64 | 947.00 | Yes |
|  | F | 59 | 4.09 | 60.8 | 95.694 | 3.00 | 93.00 | Yes |
|  | M | 56 | 2.21 | 80.3 | 94.084 | 8.11 | 91.00 | Yes |
|  | M | 20 | 5.30 | 99.0 | 94.063 | 5.80 | 227.00 | Yes |

Abbreviations: M: male; F: female; eGFR: estimated glomerular filtration rate; PLA2R: M-type phospholipase A2 receptor; RU: relative units.

eGFR levels were calculated using the Chronic Kidney Disease Epidemiology Collaboration (CKD-EPI) equation (for adults) and Schwartz equation (for children) recommended by the Kidney Disease Improving Global Outcomes (KDIGO).
