# Supplementary material for: Urine proteomics of primary membranous nephropathy using nanoscale liquid chromatography tandem mass spectrometry analysis
Source: Clin Proteomics. 2018 Feb 7;15:5. doi: 10.1186/s12014-018-9183-3 (PMC5801694; doi:10.1186/s12014-018-9183-3)
Supplement: Supplementary file 2 — Additional file 2: Table S2. Clinical characteristics of patients in group B. [file 12014_2018_9183_MOESM2_ESM.docx]

**Table S2. Clinical characteristics of patients in group B.**

|  | Gender | Age (years) | Proteinuria (g/24h) | Serum creatinine (μmol/L) | eGFR (ml/min/1.73m^2^) | Urea (mmol/L) | Serum anti-PLA2R antibody (RU/mL) | For validation |
| --- | --- | --- | --- | --- | --- | --- | --- | --- |
| *TMT1* | | | | | | | |  |
|  | F | 34 | 3.30 | 58.0 | 115.848 | 4.40 | negative | No |
|  | F | 46 | 0.64 | 37.8 | 122.589 | 2.64 | negative | No |
|  | M | 68 | 15.31 | 89.1 | 76.262 | 8.40 | negative | No |
|  | F | 54 | 1.56 | 68.0 | 87.923 | 3.76 | negative | No |
| *TMT2a* | | | | | | | |  |
|  | F | 45 | 2.85 | 49.4 | 113.048 | 5.56 | negative | No |
|  | M | 87 | 13.20 | 125.6 | 41.433 | 6.90 | negative | No |
|  | F | 40 | 2.47 | 50.2 | 116.472 | 6.47 | negative | Yes |
|  | F | 28 | 2.99 | 60.7 | 119.040 | 3.30 | negative | Yes |
|  | M | 39 | 3.25 | 76.0 | 109.247 | 4.39 | negative | Yes |
|  | F | 30 | 1.17 | 67.0 | 105.950 | 4.69 | negative | Yes |
|  | M | 23 | 0.63 | 98.5 | 92.667 | 3.96 | negative | Yes |
|  | M | 34 | 13.05 | 72.7 | 115.236 | 3.80 | negative | Yes |
|  | M | 33 | 3.39 | 81.1 | 109.264 | 4.60 | negative | No |
| *TMT2b* | | | | | | | |  |
|  | M | 56 | 2.20 | 69.1 | 100.818 | 7.49 | negative | Yes |
|  | M | 38 | 1.43 | 92.4 | 90.102 | 5.30 | negative | Yes |
|  | M | 52 | 1.72 | 43.1 | 125.891 | 4.50 | negative | Yes |
|  | F | 30 | 1.48 | 47.1 | 127.596 | 3.90 | negative | Yes |
|  | F | 16 | 7.78 | 51.0 | 115.667 | 3.82 | negative | Yes |
|  | M | 47 | 2.94 | 85.9 | 92.380 | 5.67 | negative | Yes |
|  | M | 23 | 1.82 | 61.4 | 133.444 | 5.86 | negative | No |
|  | M | 20 | 1.92 | 43.7 | 156.730 | 4.30 | negative | Yes |
|  | F | 50 | 3.78 | 39.4 | 117.578 | 3.10 | negative | No |
| *Western blot* | | | | | | | |  |
|  | F | 62 | 1.85 | 52.2 | 98.520 | 4.49 | negative | Yes |
|  | F | 53 | 1.73 | 62.6 | 97.859 | 3.80 | negative | Yes |
|  | M | 41 | 3.88 | 85.8 | 96.492 | 9.81 | negative | Yes |
|  | M | 65 | 3.36 | 116.7 | 56.205 | 8.20 | negative | Yes |
|  | M | 39 | 3.15 | 69.2 | 113.538 | 5.10 | negative | Yes |
|  | F | 54 | 3.22 | 49.9 | 105.771 | 3.85 | negative | Yes |
|  | F | 57 | 3.77 | 55.2 | 100.182 | 8.06 | negative | Yes |
|  | F | 66 | 7.06 | 169.9 | 26.711 | 7.46 | negative | Yes |
|  | M | 22 | 24.89 | 506.5 | 12.890 | 12.68 | negative | Yes |

Abbreviations: M: male; F: female; eGFR: estimated glomerular filtration rate; PLA2R: M-type phospholipase A2 receptor; RU: relative units.

eGFR levels were calculated using the Chronic Kidney Disease Epidemiology Collaboration (CKD-EPI) equation (for adults) and Schwartz equation (for children) recommended by the Kidney Disease Improving Global Outcomes (KDIGO). The results of serum anti-PLA2R antibody were considered as negative for <20 RU/mL according to the manufacturer’s protocol.
