# Supplementary material for: Urine proteomics of primary membranous nephropathy using nanoscale liquid chromatography tandem mass spectrometry analysis
Source: Clin Proteomics. 2018 Feb 7;15:5. doi: 10.1186/s12014-018-9183-3 (PMC5801694; doi:10.1186/s12014-018-9183-3)
Supplement: Supplementary file 3 — Additional file 3: Table S3. Clinical characteristics of healthy volunteers in group C. [file 12014_2018_9183_MOESM3_ESM.docx]

**Table S3. Clinical characteristics of healthy volunteers in group C.**

|  | Gender | Age (years) | Qualitative urine protein | For validation |
| --- | --- | --- | --- | --- |
| *TMT1* | | | |  |
|  | M | 26 | negative | No |
|  | M | 31 | negative | No |
|  | M | 27 | negative | No |
|  | F | 28 | negative | No |
|  | F | 28 | negative | No |
| *TMT2a* | | | |  |
|  | M | 42 | negative | Yes |
|  | F | 45 | negative | No |
|  | F | 49 | negative | No |
|  | F | 50 | negative | Yes |
|  | F | 51 | negative | No |
|  | F | 53 | negative | Yes |
|  | M | 53 | negative | Yes |
|  | M | 54 | negative | No |
|  | F | 58 | negative | No |
| *TMT2b* | | | |  |
|  | F | 45 | negative | No |
|  | M | 48 | negative | Yes |
|  | F | 50 | negative | Yes |
|  | M | 51 | negative | Yes |
|  | F | 52 | negative | No |
|  | F | 53 | negative | Yes |
|  | M | 54 | negative | Yes |
|  | F | 54 | negative | Yes |
|  | F | 43 | negative | No |
| *Western blot* | | | |  |
|  | M | 23 | negative | Yes |
|  | F | 24 | negative | Yes |
|  | M | 26 | negative | Yes |
|  | F | 21 | negative | Yes |
|  | F | 33 | negative | Yes |
|  | M | 28 | negative | Yes |
|  | M | 32 | negative | Yes |
|  | F | 29 | negative | Yes |
|  | M | 41 | negative | Yes |

Abbreviations: M: male; F: female.
